# UDP-glucose, cereblon-dependent proinsulin degrader

**DOI:** 10.1038/s41598-022-18902-5

**Published:** 2022-08-26

**Authors:** Jaeyong Cho, Atsushi Miyagawa, Kazuki Yamaguchi, Wakana Abe, Yoji Tsugawa, Hatsuo Yamamura, Takeshi Imai

**Affiliations:** 1 Department of Chemical Biology, National Center for Geriatrics and Gerontology, Obu, Aichi 474-8511 Japan; 2 Department of Life Science and Applied Chemistry, Nagoya Institute of Technology, Gokiso-cho, Showa-ku, Nagoya, 466-8555 Japan

**Keywords:** Bioinorganic chemistry, Carbohydrates, Chemical modification, Proteolysis, Endocrinology

## Abstract

Insulin secretion is regulated in multiple steps, and one of the main steps is in the endoplasmic reticulum (ER). Here, we show that UDP-glucose induces proinsulin ubiquitination by cereblon, and uridine binds and competes for proinsulin degradation and behaves as sustainable insulin secretagogue. Using insulin mutagenesis of neonatal diabetes variant-C43G and maturity-onset diabetes of the young 10 (MODY10) variant-R46Q, UDP-glucose:glycoprotein glucosyltransferase 1 (UGGT1) protects cereblon-dependent proinsulin ubiquitination in the ER. Cereblon is a ligand-inducible E3 ubiquitin ligase, and we found that UDP-glucose is the first identified endogenous proinsulin protein degrader. Uridine-containing compounds, such as uridine, UMP, UTP, and UDP-galactose, inhibit cereblon-dependent proinsulin degradation and stimulate insulin secretion from 3 to 24 h after administration in β-cell lines as well as mice. This late and long-term insulin secretion stimulation is designated a day sustainable insulin secretion stimulation. Uridine-containing compounds are designated as proinsulin degradation regulators.

## Introduction

Impairment of insulin secretion leads to diabetes mellitus^1–4^. Hypoinsulinemia in insulin variants is found in maturity-onset diabetes of the young type 10 (MODY 10) and neonatal diabetes^6–13^. Mechanistically, insulin variants in MODY10 are considered to cause diabetes due to impaired processing of insulin leading to hypoinsulinemia proinsulin^R46Q^ variant^13^ or impaired insulin receptor signaling in case of R89H-variant^14^. More severe hypoinsulinemia of the insulin variant of C43G^15^ is neonatal diabetes. The difference in MODY10 and neonatal diabetes is not yet known. Here, we found two different reasons for genetic hypoinsulinemia. One is that proinsulin^R46Q^ is retained in the endoplasmic reticulum (ER) and not secreted. The other is that proinsulin^C43G^ is ubiquitinated and degraded and then not secreted.

Insulin is secreted by stress response. One of the unfolding protein responses (UPRs) is ER-associated protein degradation (ERAD). Under (oxidative) stress, unfolding protein is eliminated by HMG-CoA reductase degradation 1 (HRD1) in the ER^16–18^. Another candidate ubiquitin ligase in the ER is cereblon^19^. Cullin–RING ubiquitin ligase complex 4 (CRL4) includes cullin4 (CUL4), DNA damage-binding protein 1 (DDB1) and cereblon (CRL4^CRBN^)^20,21^.

Here, we found that even the proinsulin^WT^ protein was also degraded by the ERAD system, indicating that inhibiting proinsulin degradation leads to insulin secretion stimulation. Insulin secretagogues, such as glucose and arginine, stimulate insulin secretion immediately and are soon reduced^22,23^. It forms one sharp peak^22,23^. Here, using inhibition of the proinsulin ERAD system, we found several chemicals that induce daily sustainable insulin secretion.

## Results

### The R46 residue of proinsulin is involved in arginine-induced insulin secretion (Figs. 1 and S1)

**Figure 1:**
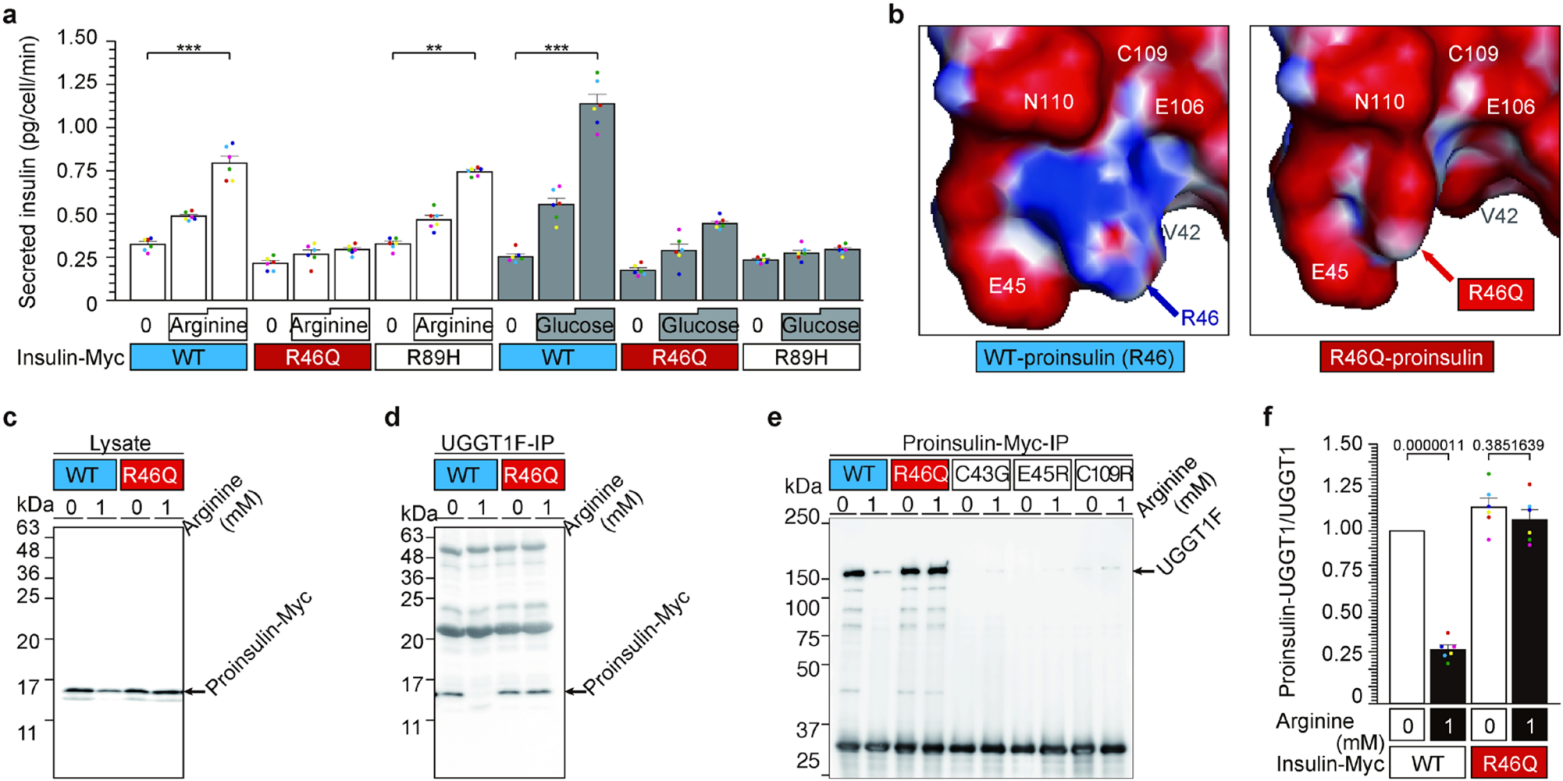
The R46 residue of proinsulin is involved in arginine-induced insulin secretion. (**a**) The R46 residue of proinsulin is involved in arginine-induced insulin secretion. NIT1 cells transfected with insulin-Myc vectors expressing WT, R46Q, and R89H were preincubated with arginine- or glucose-free F-12 K medium for 30 min. Then, the cells were treated with arginine or glucose for 5 min. The culture supernatant was collected, and secreted insulin was analyzed by ELISA. Data are presented as mean ± S.E. n = 6 **p < 0.005; ***p < 0.0001. (**b**) The predicted structure of R46 (WT) and R46Q proinsulin. Protruding R46 residue and surrounding amino acid ring of proinsulin. Basic groups are shown in blue, and acidic groups are shown in red. The original structural data of proinsulin (https://www.rcsb.org/structure/3w7y) were used ^28^. The non-protruding R46Q residue was calculated using PyMOL software. Other directions of the R46 and R46Q structures are shown in Fig. S1a. (**c**, and **d**) R46Q-mutated proinsulin protein bound to UGGT1 and was retained in the ER with/without arginine. NIT1 cells transfected with WT or R46Q proinsulin were pretreated in arginine-free F-12 k for 30 min and stimulated with 1 mM arginine for 5 min. Intracellular proinsulin and UGGT1 were analyzed by WB (**c** and Fig. S1b). Proinsulin bound to UGGT1 was assessed by IP of UGGT1 followed by IP-WB (**d** and Fig. S1c). (**e**, and **f**) Three proinsulin variants of the acid ring consisting of V42, C43, E45, E106, C109 and N110 residues of proinsulin were tested for their interaction with UGGT1 along with R46Q proinsulin. Vectors expressing mutated proinsulin were transfected, and cell lysates were prepared with or without arginine treatment. Proinsulin was immunoprecipitated and blotted for UGGT1 (**c**, **d**, Fig. S1b–d).

We previously reported that proinsulin increases its binding to UGGT1 and is retained in the endoplasmic reticulum (ER) when β-cell lines and mouse pancreases are maintained under arginine-depleted conditions^22^. Arginine administration releases proinsulin from UGGT1 in the ER and causes new complex formation between arginine and UGGT1. To assess the physiological importance of this interaction, we performed mutagenesis of the proinsulin gene to express the proinsulin variant associated with MODY 10 (maturity-onset diabetes of the young). Among four arginine residues (R46, R55, R56 and R89) in proinsulin, three variants (R46Q, R55C, R89H) are reported to cause MODY10^4–9,13,14,24–26^. R55, R56 and R89 are cleavage sites of protein convertase (PC1 and 2)^4,10,11,13,14,27^, and R46 is only one arginine residue of a non-cleavage site. Therefore, we chose two MODY 10 variants of proinsulin, R46Q and R89H, and tested the impact of the mutations on glucose- or arginine-induced secretion in NIT1 cells (Fig. 1a). Both arginine and glucose strongly induced WT insulin secretion, as expected. Glucose failed to induce secretion of MODY10 variants of R46Q and R89H insulin (Fig. 1a). Interestingly, arginine administration significantly induced R89H-insulin secretion but not R46Q-insulin secretion (Fig. 1a), indicating that the R46 residue is involved in arginine-induced insulin secretion.

The PDB database (https://www.rcsb.org/structure/3w7y)^28^ of proinsulin predicts that R46 is surrounded by a ring structure formed by valine 42 (V42), cysteine 43 (C43), glutamate 45 (E45), E106, C109, and asparagine 110 (N110) and protrudes from the plane formed by the ring (Figs. 1b and S1a). When a change in the three-dimensional structure of R46Q proinsulin was predicted using PyMOL software, the R46Q residue did not protrude from the ring (Figs. 1b and S1a). Immunoprecipitation of UGGT1 showed that the WT but not the R46 proinsulin residue interacts with UGGT1 in an arginine-dependent manner (Figs. 1c–e and S1b–d). We tested three insulin variants of C43G-neonatal diabetes, E45R and C109R, involving amino acids forming the ring structure, V42, C43, E45, E106, C109(-C43) and N110. C43G and C109R are associated with neonatal diabetes^11,14,27^. In agreement with immunoprecipitation of UGGT1, immunoprecipitation of WT insulin showed coprecipitation of UGGT1 that was lost in the presence of arginine. R46Q insulin maintains interaction with UGGT1 even in the presence of arginine. In contrast, three mutants involving the ring structure failed to interact with UGGT1 even in the absence of arginine, indicating that these residues are critical for the interaction of proinsulin with UGGT1 in the absence of arginine at the ER (Fig. 1e and S1e). Collectively, proinsulin^WT^ is bound to UGGT1 in the absence of arginine and released from UGGT1 upon arginine administration, promoting secretion. In contrast, a MODY 10 variant of proinsulin^R46Q^ is capable of binding to UGGT1 under arginine depletion but is unable to be released by arginine, preventing arginine-induced secretion of R46Q-insulin secretion.

### Impaired UGGT1-interaction in the C43G-neonatal diabetes variant (Figs. 2 and S2)

**Figure 2:**
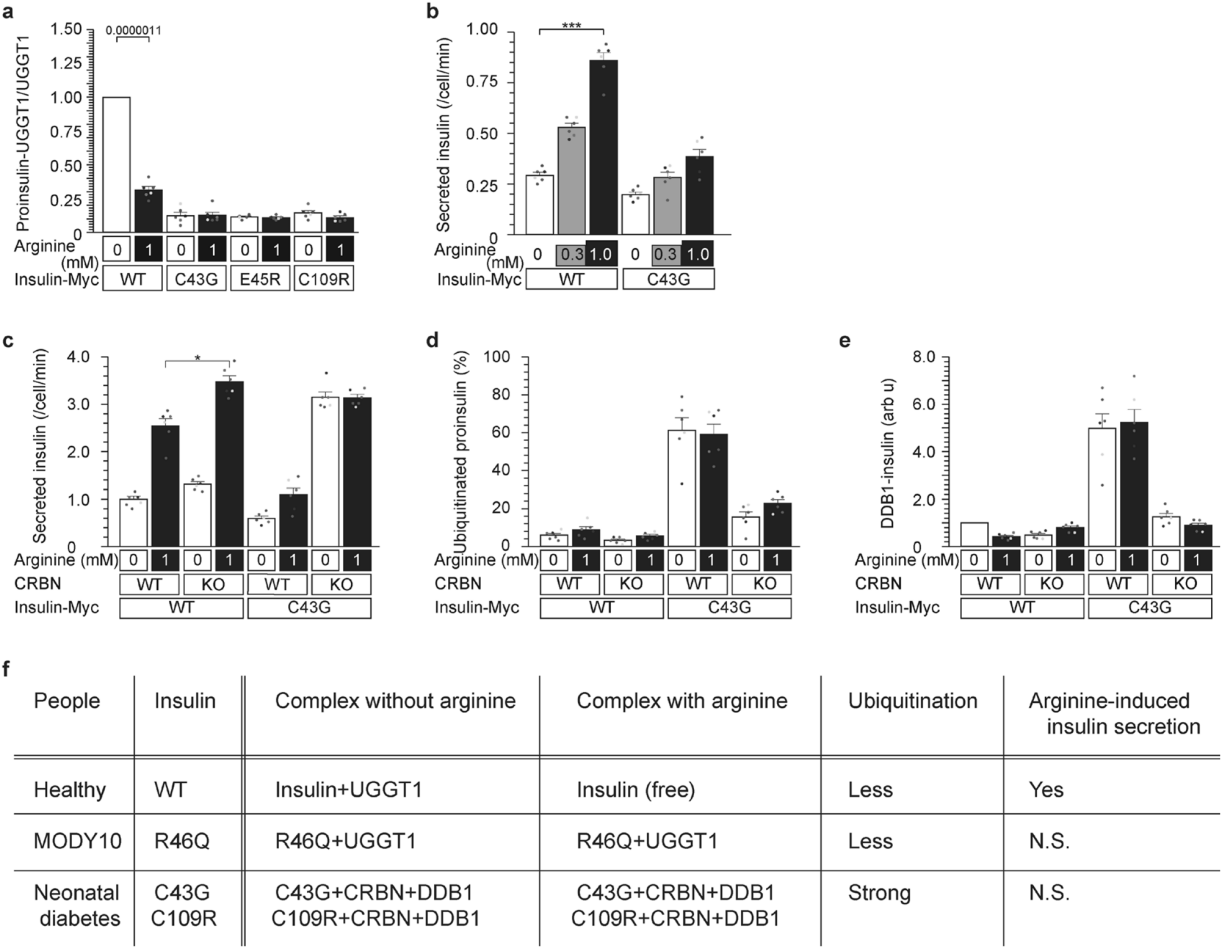
Impaired UGGT1-interaction in the C43G-neonatal diabetes variant. (**a**) Impaired interaction of mutant insulin and UGGT1. WT, C43G, E45R, and C109R insulin were analyzed for their interaction with UGGT1 by immunoprecipitation (Fig. S2a). All three variants and mutants of acidic amino acid rings failed to bind to UGGT1 in the presence or absence of arginine. (**b**–**e**) Variant MODY10^C43G^, a mutation at the acidic amino acid ring, promotes degradation by CRBN and blunts secretion in response to arginine. Arginine-induced insulin^C43G^ secretion was blunted in CRBN^WT^ and CRBN^KO^ cells (**b** and **c**). Ubiquitination of the insulin^C43G^ protein and association with DDB1 were observed only in CRBN^WT^ cells (**d**, **e**, Fig. S2a,b). (**f**) Summary of the interaction of insulin (R46Q and C43G variants) with UGGT1 and CRBN in the presence and absence of arginine.

Next, we performed further characterization of three mutants of acidic amino acids that form ring structures around the R46 residue, C43G, E45R and C109R (Figs. 1b,e and S1a,d). When proinsulin^WT^ showed binding to UGGT1 only in the absence of arginine, none of three mutants (C43G, E45R, or C109R) showed binding to UGGT1 either in the presence or absence of arginine, indicating that they may not be retained at the ER where UGGT1 resides (Fig. 2a).

We previously reported that arginine prevents the degradation of glucokinase by the E3 ubiquitin ligase cereblon (CRL4^CRBN^) using cereblon KO cells^23^. Thus, we tested whether degradation of proinsulin^C43G^ is increased and mediated by cereblon. Indeed, proinsulin^C43G^ showed ubiquitination only in NIT cells expressing cereblon (Figs. 2d and S2a). We previously reported that cereblon interacts with damage-specific DNA binding protein 1 (DDB1^20^,) for ubiquitination because they are two components of cullin ring ubiquitin ligase 4 (CRL4). In agreement with the interaction of proinsulin^C43G^ with cereblon, proinsulin^C43G^ interacted with DDB1 in cereblon^+^ cells but not in cereblon-KO cells (Figs. 2d and S2c) in the presence or absence of arginine. Then, we analyzed whether CRL4^CRBN^ contributes to arginine-induced C43G-insulin secretion (Figs. 2b–d and S2a,b). The secretion of C43G mutant insulin in response to arginine was blunted compared with WT insulin in NIT1 cells expressing cereblon (Fig. 2b,c). The impairment in arginine-induced insulin secretion was not rescued by cereblon knockout in NIT1 cells expressing the C43G variant (Figs. 2c and S2b).

Collectively, these data indicated that proinsulin^C43G^ interacts with cereblon and DDB1 instead of UGGT1, which leads to ubiquitination of proinsulin^C43G^ by cereblon and subsequent degradation (Fig. 2e). Both proinsulin^WT^ and proinsulin^R46Q^ interact with UGGT1 in the absence of arginine (Fig. 2e). Whereas proinsulin^WT^ is released from UGGT1 and secreted when arginine becomes available, the R46Q variant does not compete with arginine and remains bound to UGGT1, leading to ER retention and reduced secretion (Fig. 2e).

Based on two mutants, we propose that R46 and C43 residues of proinsulin have a distinct role in mediating arginine-induced insulin secretion. The R46 residue competes with arginine for the interaction with UGGT1, and the C43 residue mediates binding regardless of the availability of arginine. Thus, two variants (R46Q and C43G) responsible for MODY and neonatal diabetes cause impaired arginine-induced insulin secretion due to altered interaction with UGGT1.

### Arginine switches proinsulin associated E3 ubiquitin ligases from HRD1 to cereblon (Figs. 3 and S3)

**Figure 3:**
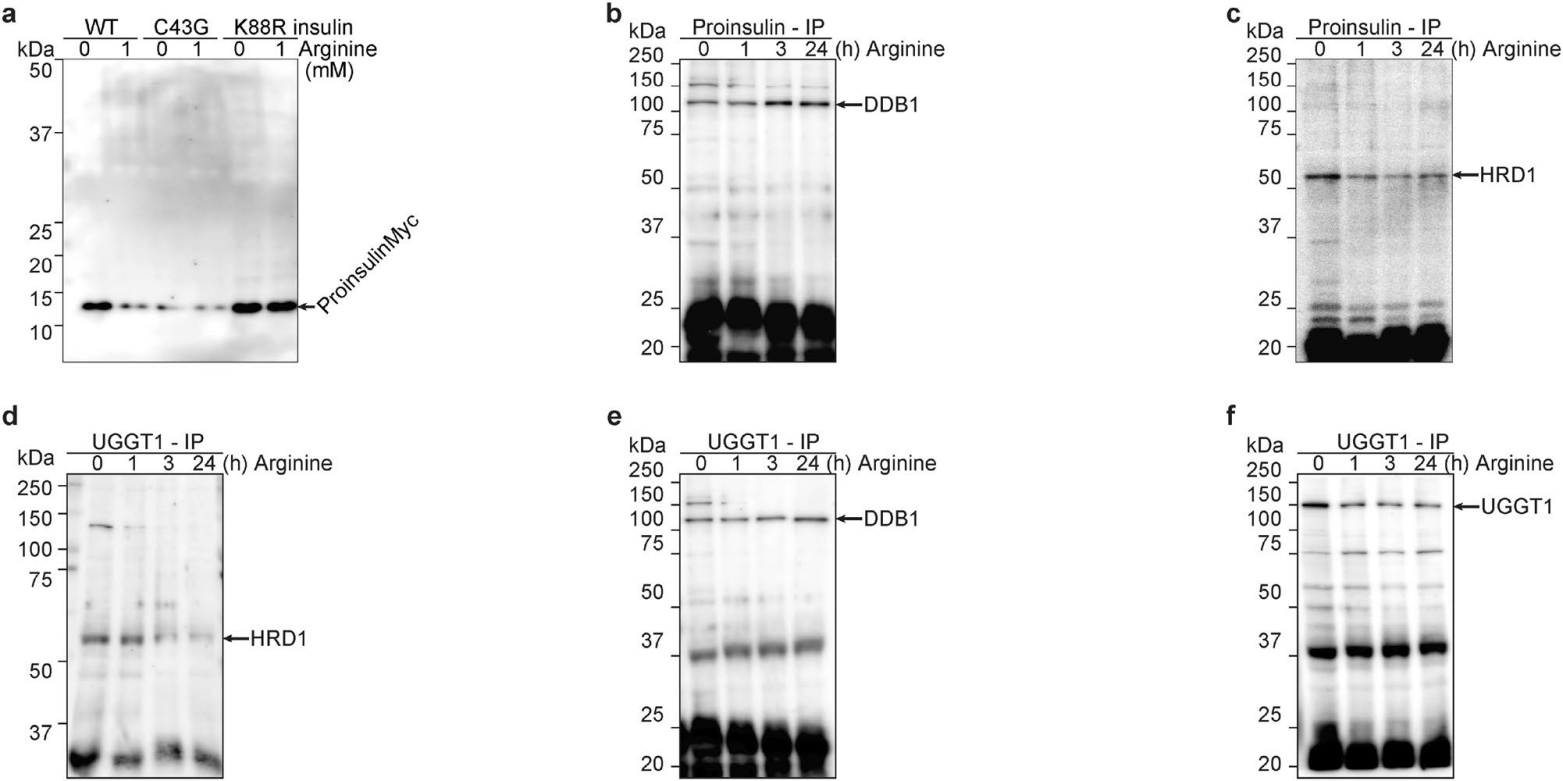
Arginine switches proinsulin associated E3 ubiquitin ligases from HRD1 to CRBN. (**a**) WT proinsulin was degraded after arginine administration, and C43G proinsulin was degraded with/without arginine, although K88R proinsulin was not degraded with/without arginine. Arginine was depleted for 30 min, and cells were treated with 0 or 1 mM arginine. The cell lysates were visualized with anti-Myc WB. (**b**–**f**) Both proinsulin and UGGT1 are associated with the E3 ubiquitin ligase DDB1 after arginine administration. Arginine was depleted from the medium for 30 min, and NIT1 cells were treated with 1 mM arginine for the indicated times (0, 1, 3, and 24 h). The cell lysates were IPed with proinsulin (**b** and **c**) or UGGT1 (**d**–**f**), and then these IP-ed lysates were visualized with DDB1 (**b**, and **e**), HRD1 (c and d), or UGGT1 (**f**, control).

In Fig. 2, we demonstrate that UGGT1 not only retains proinsulin in the ER but also protects proinsulin from ubiquitination and degradation by CRL4^CRBN^. This protection is releasing by arginine-replacing. Ubiquitination occurs on the lysine residue of K88-proinsulin. Non-ubiquitinated K88R-proinsulin actually existed at much higher levels than WT-proinsulin (Fig. 3a). This indicates that WT proinsulin in the ER is ubiquitinated and degraded. Non-ubiquitinated proinsulin K88R was secreted much more highly than WT proinsulin (Fig. S3a).

Proinsulin is one of the forms of insulin in the ER and the Golgi body^1,2^, leading to proinsulin ubiquitination called ERAD (ER-associated degradation). The main E3 ubiquitin ligase in ERAD is thought to be HRD1 (HMG-CoA reductase degradation protein 1)^16–18^. Other E3 ubiquitin ligases, CRL4 (CUL4-DDB1-CRBN), also include proinsulin ubiquitination (Fig. 2). First, arginine-induced proinsulin ubiquitination enzymes were investigated (Fig. 3b,c). Arginine administration induces proinsulin-associated DDB1 (Fig. 3b); nevertheless, arginine administration reduces proinsulin-associated HRD1 (Fig. 3c). On the other hand, UGGT1-associated E3 ubiquitin ligases were also analyzed. UGGT1 was also associated with DDB1 after arginine administration (Fig. 3d), but HRD1 dissociated after arginine administration (Fig. 3e). In fact, proinsulin and UGGT1 were mainly degraded after arginine administration (Fig. 3a and f), indicating that CRL4 (CUL4-DDB1-CRBN) is the E3 ubiquitin ligase that ubiquitinates and degrades proinsulin and UGGT1 after arginine administration. Cereblon colocalizes with UGGT1 in the ER with UDP-glucose and arginine (Fig. S3b).

### Uridine and succinimide protect proinsulin degradation from cereblon and stimulate insulin secretion from 3 to 24 h (Figs. 4 and S4)

**Figure 4:**
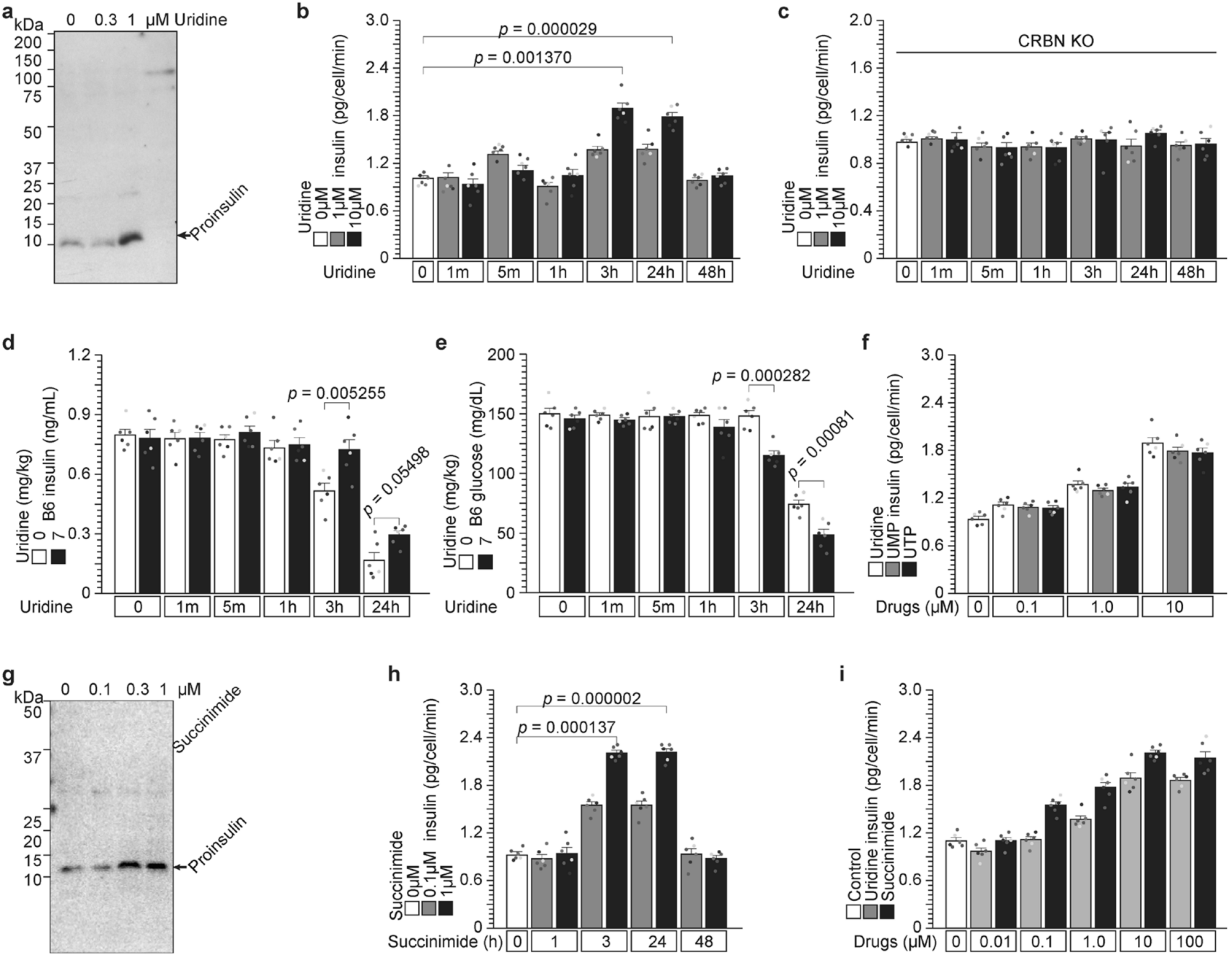
Uridine and succinimide protect proinsulin degradation from cereblon and stimulate insulin secretion from 3 to 24 h. (**a**–**e**) Uridine binds to cereblon and protects against proinsulin degradation by endogenous ligand X. (**a**) Uridine protects against proinsulin degradation in a dose-dependent manner. NIT1 cells were treated with 0, 0.3, or 1 µM uridine for 3 h. Intracellular proinsulin was detected by WB. (**b** and **c**) Uridine administration to NIT1 cells stimulates insulin secretion from 3 to 24 h with a cereblon contribution. Uridine was administered to CRBN-WT (**b**) and CRBN-KO (**c**) cells, and insulin secretion was analyzed using ELISA from 1 m to 48 h. (**d** and **e**) Uridine (7 mg/kg) administration to C57/BL6J mice induces circulating insulin (**d**) and reduces circulating glucose (**e**) from 1 m to 24 h. (**f**) Other uridine-containing compounds, UMP and UTP, stimulate insulin secretion from 3 to 24 h. (**g**–**i**) Succinimide also binds to cereblon and protects against proinsulin degradation. (**g**) Succinimide protects against proinsulin degradation in a dose-dependent manner. NIT1 cells were treated with 0, 0.1, 0.3, and 1 µM succinimide for 3 h. (**h** and **i**) Succinimide administration to NIT1 cells stimulated insulin secretion from 3 to 24 h in a dose-dependent manner.

Therefore, we demonstrate that UGGT1 protects against proinsulin ubiquitination by CRL4 (CUL4-DDB1-CRBN), and arginine administration releases this protective effect (Fig. 3), indicating that arginine-induced insulin secretion is a new step in inducing proinsulin degradation. If, this degradation is reduced, stimulating insulin secretion as a drug target such as K88R-insulin (Figs. 3a and S3a).

How repress cereblon-dependent ubiquitination? Cereblon is ligand-induced E3 ubiquitin ligase. First identified exogenous ligand is thalidomide^20^. Its substrate (ubiquitinated) protein is Ikaros^26^. A possible endogenous ligand is uridine^24^, but its substrate protein is not known. MEIS2 has been identified as an unknown endogenous/natural ligand A by competing with thalidomide^26^. Another exogenous ligand is succinimide^24^. cereblon-binding chemicals, such as uridine and succinimides, bind to cereblon and compete for endogenous/natural ligand A or proinsulin degrader X (Fig. S4c).

First, we administered uridine to NIT1 cells (Fig. 4a–c) and C57BL/6 J mice (Fig. 4d,e). Uridine released proinsulin degradation by endogenous proinsulin degrader X and increased proinsulin protein levels in a dose-dependent manner (Fig. 4a). Surprisingly, uridine-induced insulin secretion occurs from 3 to 24 h (Fig. 4b), because general insulin secretions, such as glucose and arginine, induce insulin secretion in minutes^22,23^. This induction was not observed after 1–5 min or in cereblon KO cells (Fig. 4b,c), indicating that uridine-competitive proinsulin degradation occurs through CRL4^CRBN^. Uridine administration to C57BL6 mice also induced insulin and reduced glucose concentrations from 3 to 24 h (Fig. 4d,e). There was no insulin induction immediate early of 1 and 5 min after administration in cells as well as WT-mice (Fig. 4b–e). Taken together, uridine-induced insulin secretion starts late and sustainably. Note that other uridine-containing chemicals, UMP and UTP, also induced insulin secretion (Fig. 4f) sustainably.

We have used CRBN^YWAA^ mutant without thalidomide binding activity (^1^ and Fig. S4a). Glutarimides of immunomodulatory drugs (IMiDs), thalidomide, lenalidomide, and pomalidomide, bind to cereblon, although IMiDs do not bind to cereblon^YWAA^ (Fig. S4a). IN fact, uridine and UDP-glucose contain glutarimide structure (Fig. S4a). So, using CRBN^YWAA^ mutant, UDP-glucose dependent UGGT1 degradation was analyzed, and CRBN^YWAA^ mutant protein was resistant UDP-glucose triggering UGGT1 degradation (Fig. S4b,c). UDP-glucose bound to cereblon similar way to IMiDs, and induced UGGT1 degradation.

Succinimide (Fig. 4g–i) is an artificial cereblon-binding chemical^24^ and is a smaller molecule than uridine. Succinimide might not have a substrate protein-binding motif and has a higher affinity for cereblon than uridine^24^, indicating that succinimide may behave as a stronger cereblon inhibitor and stimulate insulin secretion than uridine. In fact, succinimide binds to cereblon, protects proinsulin degradation from proinsulin degrader X in a dose-dependent manner (Fig. 4g), and sustainably stimulates insulin secretion (Fig. 4h). Succinimide stimulates insulin secretion more than uridine does (Fig. 4i). The IC_50_ of succinimide was 1.541 μM (Fig. S4a), and the IC_50_ of uridine was 0.3473 μM (Fig. S4b).

### UDP-glucose is an endogenous proinsulin protein degrader through cereblon (Figs. 5 and S5)

**Figure 5:**
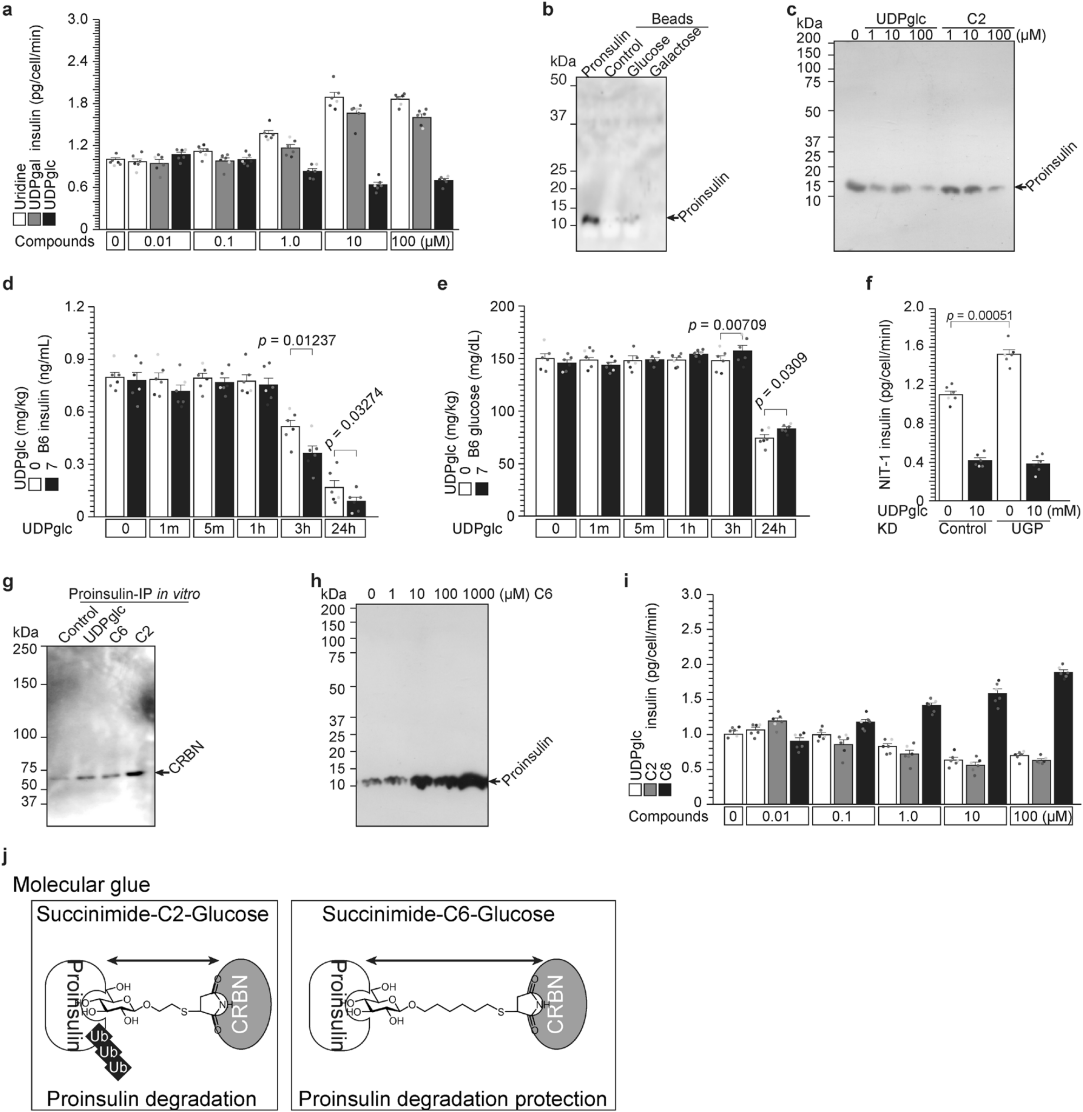
UDP-glucose is an endogenous proinsulin protein degrader through cereblon. (**a**–**c**) The proinsulin protein degrader X is UDP-glucose. Uridine and UDP galactose administration increased insulin secretion (Fig. 4), although UDP glucose decreased insulin secretion in a dose-dependent manner (a). Proinsulin binds to glucose but does not bind to galactose (**b**). UDP glucose and succinimide-C2-glcuose degraded proinsulin protein (**c**). (**d** and **e**) Administration of the proinsulin protein degrader UDP-glucose to C57/BL6J mice decreased circulating insulin (**d**) and increased glucose concentration (**e**). (**f**) Significant higher insulin secretion in UGP KD cells^31^. (**g**–**j**) Succinimide-glucose degrades or protects proinsulin. (**g**) Succinimide-C2-glucose strongly stimulates the proinsulin and cereblon interaction in vitro. (**h**) Succinimide-C6-glucose protects endogenous UDP-glucose-dependent proinsulin degradation in a dose-dependent manner. (**i**) UDP glucose and succinimide-C2-glucose reduced insulin secretion in a dose-dependent manner, although succinimide-C6-glucose induced insulin secretion (like UDP-galactose). (**j**) Possible model of molecular glue with/without proinsulin degradation.

We explored uridine monosaccharide to identify the proinsulin protein degrader X. Similar to uridine (Fig. 4), UDP-galactose also stimulated insulin secretion in β cells (Fig. 5a), while UDP-glucose repressed insulin secretion (Fig. 5a). These data indicated that UDP-glucose is the proinsulin degrader X, which degraded proinsulin in a dose-dependent manner. Uridine binds to cereblon (Fig. 4), and glucose weakly binds to proinsulin (Fig. 5b). Nevertheless, galactose does not bind to proinsulin (Fig. 5b). These data suggested that UDP-glucose combined with CRL4^CRBN^ and proinsulin as a molecular glue. UDP-glucose administration to NIT1 cells reduced proinsulin in a dose-dependent manner (Figs. 5c and S5b–d, IC50 = 1.322 μM). UDP-glucose administration to C57/BL6J mice reduced insulin concentrations (Fig. 5d) and induced glucose concentrations (Fig. 5e) from 3 to 24 h, not immediate early times of 1 and 5 min. Furthermore, uridine-induced insulin secretion and UDP-glucose-reduced insulin secretion were confirmed with high glucose condition of NIT-1 (Fig. S5k), MIN6, and RIN5-F cells (Fig. S5l).

### UDP-glucose and cereblon degrade UGGT1 (Figs. 5 and S5)

Cereblon degrade UGGT1 as well (Figs. 3 and S3). UDP-glucose is also UGGT1 degrader. I*n vitro* IP-WB was performed (Fig. S5a). Protein–protein interaction was observed without UDP-glucose, and UDP-glucose induced the interaction (Fig. S5a). Using KD of cereblon and HRD1, and cereblon concerned with UDP-glucose dependent UGGT1 degradation (Fig. S5b).

We used two mutant proteins (Fig. S5c), and one is GTF which defect glucose transfer activity^29^. CRBN^YWAA^ mutant protein has less binding activity to thalidomide^1^ and as well as uridine (Fig. S4a–c). Near 384Y and 386 W, there are two tryptophan residues of 380 W and 400 W. These three tryptophan residues are indispensable for IMiDs binding^30^. In the UGGT1, we found similar three tryptophan region, and made mutant named UGGT1^3W>3A^ (Fig. S5c). Uridine or glucose binding activity of these three UGGT1 proteins were analyzed, and indicating that WT and GTF mutant proteins bound to uridine as well as glucose, although 3 W > 3A mutant did not bind neither uridine nor glucose (Fig. S5d). Cereblon-UGGT1 interaction was analyzed, and UDP-glucose induced interaction of CRBN^WT^ (Fig. S5a and e), although did not induce interaction of CRBN^3W>3A^ (Fig. S5e,f).

Intracellular UDP-glucose concentration of various cells was analyzed by metabolome analysis (Fig. S5g). The highest UDP-glucose concentration was in HEK293 cells and the lowest concentration in NCI-H929-1α cells. UDP-glucose was administered to these cells and the UGGT1 degradation was analyzed (Fig. S5i,j). Higher UDP-glucose (1 mM) was necessary to HEK293, and lower UDP-glucose (100 μM) was necessary for H929-1α cells.

UDP-glucose pyrophosphorylase (UGP) is the enzyme of UDP-glucose synthesis from glucose-1-phosphate (G1P) and UTP.

$$ \hbox{``}G1P{\text{ }} + {\text{ }}UTP \rightleftharpoons UDP - glu\cos e{\text{ }} + {\text{ }}pyrophosphate\hbox{''}  $$

UGP was knocked down (KD) in NIT-1 cells^31^. UDP-glucose was administered to these cells, and insulin secretion was analyzed (Fig. 5f). Significantly higher insulin secretion was observed in UGP-KD cells, but secreted insulin reduction by UDP-glucose administration were similarly observed in control and UGP-KD NIT-1 cells (Fig. 4f). Taken together, UDP-glucose administration in pancreatic β-cells first induces an interaction between cereblon and proinsulin as the molecular glue and then induces ubiquitination and degradation of proinsulin. Degraded proinsulin also reduced insulin secretion.

### UDP-glucose and succinimide-glucose degrade insulin (Figs. 5 and S5)

Succinimide binds to cereblon with higher affinity than uridine (Fig. 4, and^24^), indicating that succinimide-linker-glucose may behave as a proinsulin degrader. We synthesized two linker lengths (C_2_H_4_ and C_6_H_12_), and the linkers were combined with succinimide and glucose. Succinimide-C2-glucose (C2) and succinimide-C6-glucose (C6) were synthesized and analyzed. In vitro complex formation analysis showed that UDP-glucose and C6 stimulated complex formation rather weakly, and C2 stimulated complex formation strongly (Fig. 5g). UDP-glucose and C2 degraded proinsulin in a dose-dependent manner (Fig. 5c), although surprisingly, C6 induced proinsulin protein in a dose-dependent manner (Fig. 5h). These phenomena are reproducible for insulin secretion (Fig. 5i). UDP-glucose and C2 reduce insulin secretion, although C6 induces insulin secretion in a dose-dependent manner. Note: UDP-glucose-IC_50_ = 1.322 μM (Fig. S5b–d), C2-IC_50_ = 0.475 μM (Fig. S5e–g), C6-IC_50_ = 0.489 μM (Fig. S5h-j).

## Discussion

### Proinsulin ERAD by cereblon controlled by uridine and UDP-glucose

ER-associated protein degradation (ERAD) is one unfolded protein response (UPR) cascade that eliminates unfolded proteins. Generally, unfolded proteins in the ER are ubiquitinated by HRD1. The HRD1 ubiquitination system is controlled by oxidative stress. Here, we identify other E3 ubiquitin ligase CRL4^CRBN^. Cereblon is ligand-inducible E3 ubiquitin ligase. The first identified ligand is thalidomide, and the thalidomide-CRBN substrate protein is Ikaros^21^. In addition, we identified two types of CRBN ubiquitination signaling molecules: agonists (UDP-glucose) and antagonists (uridine). Both are endogenous chemicals that always exist in each cell. This indicates endogenous chemical control of the ERAD system. As described above, the existence of unfolding proteins triggers ERAD but does not control other molecules. This is the first evidence of endogenous chemicals controlling UPR and ERAD.

### UDP-glucose-dependent proinsulin degradation

Not only unfolded proinsulin^C43G^ mutated protein but also proinsulin^WT^ protein were degraded by cereblon (Figs. 3 and 4). Endogenous cereblon ligand for proinsulin degradation is UDP-glucose (Fig. 5). So, most probably proinsulin is always degraded by endogenous UDP-glucose. Using UGP KD system, endogenous UDP-glucose was decreased^31^. Insulin secretion were increased by MGP-KD (Fig. 5f). The data indicated that endogenous UDP-glucose degraded proinsulin protein. Administrated uridine competed with endogenous UDP-glucose-dependent proinsulin degradation, and led to increasing insulin secretion (Fig. 4 and S4). So, not only uridine (Figs. 4a–f,i, S4d and S5j,k), but also administered UMP (Fig. 4f), UTP (Fig. 4f), and UDP-galactose (Fig. 5a,b) inhibited UDP-glucose-dependent proinsulin degradation.

### Possible physiological role of UDP-glucose-dependent proinsulin degradation

**Figure 6:**
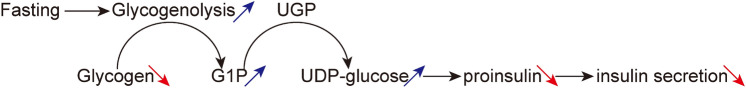
Possible model of fasting induced UDP-glucose- and cereblon-dependent proinsulin degradation.

UDP-glucose is produced from G1P and UTP by UDP-glucose pyrophosphorylase (UGP, Fig. 5, and ^31^), and G1P is produced from glycogen by glycogenolysis ^32^. Glycogenolysis is the breakdown of glycogen (n) to G1P and glycogen (n-1). Glycogenolysis is mainly activated by fasting ^32^. Physiological role of UDP-glucose-induced proinsulin degradation might be one of the signaling under fasting (Fig. 6).

### Preclinical study

Xu et al.(2018) reported preclinical study, using Akita mice with INS2^C96Y/C96^ (INS1^C96/C96^,^33^. This mouse insulin mutant disease is similar to rare disease MODY10 variant, dominant mutation. Here, we used insulin^WT/WT^, might be similar to type2 DM or aged people. Mutated protein (INS2^C96Y^) is generally recognized unfolded protein response (UPR), and eliminated with ER-associated protein degradation (ERAD) system with HRD1. Here, we identified other E3 ubiquitin ligase cereblon with UDP-glucose triggering.

## Conclusion

We previously reported that proinsulin is retained in the ER through binding to the ER resident protein UGGT1 when the availability of arginine is low and that arginine releases proinsulin from the ER through competition with UGGT1^22^. Here, we report MODY10- or neonatal diabetes-associated proinsulin variants that interfere with their interaction with UGGT1. Through the analysis of these variants, we further refined the molecular basis of the interaction between proinsulin and UGGT1. Proinsulin binds to UGGT1 via the R46 residue and amino acid residues that form a ring around R46 when the availability of arginine is low in the ER, causing the retention of proinsulin in the ER of pancreatic β cells. The C43, E45, and C109 residues of proinsulin form a ring structure that mediates the binding of proinsulin to UGGT1. In support of this hypothesis, C43G and C109R variants of proinsulin associated with neonatal diabetes failed to bind UGGT1. These variants bind the CRL4 E3 ubiquitin ligase of cereblon and DDB1 instead, leading to degradation after ubiquitination both in the presence and absence of arginine. This allows proinsulin to be further processed at the Golgi network and mature into secretory vesicles for insulin secretion. Proinsulin^R46Q^ and proinsulin^C43G^ genetic mutations associated with MODY/neonatal diabetes all cause impairment in arginine-inducing insulin secretion but cause distinct defects in the interaction between UGGT1 and proinsulin.

Free proinsulin^WT^ also interacts with UDP-glucose and CRBN + DDB1 after dissociation from UGGT1 and is ubiquitinated. UDP-glucose and proinsulin are the first identified endogenous cereblon ligands and their substrate proteins.

This UDP-glucose-dependent proinsulin degradation is associated with linker length and is important. A shorter linker (C2) strengthens the complex, and proinsulin is degraded. A longer linker (C6) makes the complex weaker, but proinsulin is not ubiquitinated.

Using uridine or succinimide, proinsulin degradation by UDP-glucose and cereblon was inhibited, and insulin secretion was induced from 3 to 24 h (Fig. 4). Generally, insulin secretion stimulation is in minutes^22,23^. Insulin secretagoues (glucose, arginine, etc.) activate glucokinase activity and UGGT1 ER retention and stimulate insulin secretion immediately. Instead of acute induction by glucose or arginine, uridine, succinimide and succinimide-C6-glucose stimulation starts late and sustainably. They might be developing a new anti-diabetes drug.

## Methods

### Antibodies

The following antibodies were purchased: β-actin (sc-47778, Santa Cruz Biotechnology, Santa Cruz, CA, USA), insulin (L6B10, Cell Signaling Technology, Danvers, MA, USA, and sc-9168, Santa Cruz Biotechnology), UGGT1 (14,170–1-AP, Proteintech, Rosemont, IL, USA; sc-374565, Santa Cruz Biotechnology; and ab13520-50, Abcam), and FLAG (F-1804, Sigma, St. Louis, MO, USA). UGP knockdown siRNA (sc-154894) was purchased from Santa Cruz^31^.

### Cells

Mouse β NIT-1 cells (CRL-2055TM purchased from ATCC, Manassas, VA, USA), human embryo kidney 293 T (HEK293T) cells and human hepatocellular carcinoma (Hep G2) cells were maintained as described previously^16,17,22,23,25,26,34–36^.

### Analysis of insulin secretion from cells

Insulin secretion was determined using a commercial enzyme-linked immunosorbent assay (ELISA) kit (Shibayagi, Gunma, Japan)^22,23^ as described previously.

### Immunoprecipitation (Ip) and Western blot (WB)

Ip and WB analysis were performed as described previously^22,23,25,26,34–36^. Immune complexes were detected using Immunostar LD (Wako) substrate. Signals were quantified with the LAS 4000 imaging system (GE Healthcare).

### Structure analysis

Homology models of human proinsulin were generated using the Swiss-Model server. The template with the highest quality (2kqp.1; PDB ID: 2KQP)^28^ was selected for model building. Computer modeling of R46Q was performed using the PyMOL mutagenesis algorithm wherein the backbone-dependent rotamers of glutamine were examined. Only the rotamer with the highest frequency of appearance in proteins is shown. Global and per-residue model quality was evaluated using the QMEAN4 scoring function. The obtained scores were –5.83 and –6.06 for wild-type and mutant humans, respectively.

### Proinsulin mutants

Using mutated synthesized oligomers and a PrimeSTAR mutagenesis basal kit (Takara, Kyoto, Japan), mutated insulin and UGGT1 expression vectors were produced. NIT-1 cells were transfected with pCDNA-insulin (WT, C43G, E45R, R46Q or C109R)-Myc expression vectors with Lipofectamine 2000 (Thermo Fisher, Rockford, IL, USA). On the next day, these cells were cultivated in arginine-free medium for 30 min before arginine was added. The supernatants were analyzed with a Myc-ELISA kit (Shibayagi, Japan). The transfection efficiency was ~ 10% for NIT-1 cells and 80% for HEK293FT cells.

### Chemical synthesis

#### General

^1^H and ^13^C NMR spectra were recorded in CDCl_3_-d and D_2_O using a Bruker AVANCE 400 Plus Nanobay instrument (400 and 101 MHz) and a Bruker AVANCE 500 instrument fitted with a cryoprobe (500 and 126 MHz), respectively. Chemical shifts (δ) are given in ppm and referenced to tetramethylsilane (0.00 ppm) or the internal solvent signal used as an internal standard. Assignments in the NMR spectra were made by first-order analysis of the spectra and were supported by ^1^H − ^1^H COSY and ^1^H − ^13^C HMQC correlation results. Matrix-assisted laser desorption ionization time-of-flight high-resolution mass spectrometry (MALDI-TOF HRMS) spectra were recorded on a Jeol JMS-S3000 using 2,5-dihydroxylbenzoic acid as the matrix. Unless otherwise stated, all commercially available solvents and reagents were purchased from FUJIFILM Wako Pure Chemical Corporation and Tokyo Chemical Industry Company Limited without further purification.

#### 2-Bromoethyl 2,3,4,6-tetra-O-acetyl-β-D-glucopyranoside (2)

To a solution of compound 1 (3.00 g, 6.09 mmol) and 2-bromoethanol (743 μL, 10.4 mmol) in dichloromethane (28.4 mL) was added molecular seives 3 Å powder (3.00 g), and cooled at − 40 °C. To the mixture was added boron trifluoride-ethyl ether complex (229 μL, 1.82 mmol) diluted in dichloromethane (2.1 mL) and stirred at − 40 °C for 30 min. The solution was diluted with dichloromethane and filtered through celite. The filtrate was washed with aqueous sodium hydrogen carbonate and brine, dried over anhydrous sodium sulfate, filtered, and evaporated. The residue was purified by silica gel chromatography with 8:1 to 4:1 (v/v) hexane–ethyl acetate to give 2 (1.84 g, 66%): 1H NMR (CDCl3, 400 MHz) δ 5.22 (t, 1 H, J2,3 = 9.5 Hz, H-3), 5.09 (t, 1 H, J3,4 = 9.5 Hz, H-4), 5.02 (dd, 1 H, Hz, H-2), 4.58 (d, 1 H, J1,2 = 8.0 Hz, H-1), 4.58 (dd, 1 H, J = 4.8 Hz, J6a,6b = 12.3 Hz, H-6a), 4.20–4.13 (m, 2 H, H-6b, –OCH_2_–), 3.85–3.79 (m, 1 H, –OCH_2_–), 3.74–3.69(m, 1 H, H-5), 3.49–3.45 (m, 2 H, –CH2Br), 2.10 (s, 3 H, Ac), 2.08 (s, 3 H, Ac), 2.03 (s, 3 H, Ac), 2.02 (s, 3 H, Ac); 13C{1H} NMR δ (CDCl3, 101 MHz): 170.6, 170.2, 169.4, 169.4, 101.0, 72.6, 71.9, 71.0, 69.8, 68.3, 61.8, 29.8, 20.7, 20.6, 20.5.

#### 6-Bromohexyl 2,3,4,6-tetra-O-acetyl-β-D-glucopyranoside (3)

To a solution of compound 1 (3.00 g, 6.09 mmol) and 6-bromoethanol (2.49 mL, 10.3 mmol) in dichloromethane (28.4 mL) was added molecular sieves 4 Å powder (3.00 g) and cooled at − 40 °C. To the mixture was added boron trifluoride-ethyl ether complex (229 μL, 1.82 mmol) diluted in dichloromethane (2.1 mL) and stirred at − 40 °C for 30 min, at − 20 °C for 30 min and then at 0 °C for 30 min. The solution was diluted with dichloromethane and filtered through celite. The filtrate was washed with aqueous sodium hydrogen carbonate and brine, dried over anhydrous sodium sulfate, filtered, and evaporated. The residue was purified by silica gel chromatography with 10:1 to 3:1 (v/v) hexane–ethyl acetate to give 3 (2.06 g, 66%): 1H NMR (CDCl3, 400 MHz) δ 5.21 (t, 1 H, J2,3 = 9.5 Hz, H-3), 5.09 (t, 1 H, J3,4 = 9.7 Hz, H-4), 4.99 (dd, 1 H, Hz, H-2), 4.49 (d, 1 H, J1,2 = 8.0 Hz, H-1), 4.27 (dd, 1 H, J = 4.7 Hz, , J6a,6b = 12.3 Hz, H-6a), 4.14 (dd, 1 H, J5,6a = 2.4 Hz, H-6b), 3.91–3.85 (m, 1 H, − OCH_2_− ), 3.72–3.67 (m, 1 H, H-5), 3.51–3.46 (m, 1 H, − OCH_2_− ), 3.41 (t, 2 H, J = 6.8 Hz, − CH2Br), 2.11 (s, 3 H, Ac), 2.05 (s, 3 H, Ac), 2.03 (s, 3 H, Ac), 2.01 (s, 3 H, Ac), 1.89–1.82 (m, 2 H, − CH_2_− ), 1.62–1.55 (m, 2 H, − CH_2_− ), 1.46–1.32 (m, 4 H, − CH_2_− ); 13C{1H} NMR (CDCl3,101 MHz) δ 170.7, 170.3, 169.4, 169.2, 100.8, 72.8, 71.7, 71.3, 69.9, 68.4, 62.0, 33.7, 32.6, 29.2, 27.7, 25.0, 20.7, 20.6, 20.6, 20.6.

#### 2-S-Thioacetylethyl 2,3,4,6-tetra-O-acetyl-β-D-glucopyranoside (4)

To a solution of compound 2 (500 mg, 1.10 mmol) and thioacetic acid (117 μL, 1.64 mmol) in N,N-dimethylformamide (3.67 mL) was added potassium carbonate (228 mg, 1.65 mmol) and stirred at room temperature for 12 h. The solution was diluted with ethyl acetate, washed with aqueous sodium hydrogen carbonate and brine, dried over anhydrous sodium sulfate, filtered, and evaporated. The residue was purified by silica gel chromatography with 4:1 (v/v) hexane–ethyl acetate to give 4 (492 mg, 99%): 1H NMR (CDCl3, 400 MHz) δ 5.21 (t, 1 H, J2,3 = 9.5 Hz, H-3), 5.09 (t, 1 H, J3,4 = 9.7 Hz, H-4), 4.99 (dd, 1 H, Hz, H-2), 4.53 (d, 1 H, J1,2 = 8.0 Hz, H-1), 4.26 (dd, 1 H, J = 4.8 Hz, , J6a,6b = 12.3 Hz, H-6a), 4.14 (dd, 1 H, J = 2.4 Hz, , J6a,6b = 12.3 Hz, H-6b), 4.01–3.95 (m, 1 H, –OCH_2_–), 3.73–3.68 (m, 1 H, H-5), 3.65–3.59 (m, 1 H, H-5, –OCH_2_–), 3.26–3.01 (m, 2 H, –CH_2_S), 2.34 (s, 3 H, SAc), 2.09 (s, 3 H, Ac), 2.08 (s, 3 H, Ac), 2.03 (s, 3 H, Ac), 2.01 (s, 3 H, Ac); 13C{1H} NMR (CDCl3, 101 MHz) δ 195.2, 170.6, 170.2, 169.4, 169.3, 100.8, 72.7, 71.9, 71.1, 68.6, 68.3, 61.8, 30.5, 28.8, 20.7, 20.7, 20.6, 20.5.

#### 6-S-Thioacetylhexyl 2,3,4,6-tetra-O-acetyl-β-D-glucopyranoside (5)

To a solution of compound 3 (700 mg, 1.37 mmol) and thioacetic acid (146 μL, 1.79 mmol) in N,N-dimethylformamide (4.57 mL) was added potassium carbonate (284 mg, 2.05 mmol) and stirred at room temperature for 17.5 h. The solution was diluted with ethyl acetate, washed with water and brine, dried over anhydrous sodium sulfate, filtered, and evaporated. The residue was purified by silica gel chromatography with 8:1 to 4:1 (v/v) hexane–ethyl acetate to give 5 (561 mg, 83%): 1H NMR (CDCl3, 400 MHz) δ 5.20 (t, 1 H, J2,3 = 9.5 Hz, H-3), 5.09 (t, 1 H, J3,4 = 9.7 Hz, H-4), 4.98 (dd, 1 H, Hz, H-2), 4.49 (d, 1 H, J1,2 = 8.0 Hz, H-1), 4.27 (dd, 1 H, J = 4.7 Hz, , J6a,6b = 12.3 Hz, H-6a), 4.14 (dd, 1 H, J = 2.4 Hz, J6a,6b = 12.3 Hz, H-6b), 3.90–3.84 (m, 1 H, –OCH_2_–), 3.71–3.67 (m, 1 H, H-5), 3.50–3.44 (m, 1 H, H-5, –OCH_2_–), 2.85 (t, 2 H, J = 7.3 Hz –CH_2_S), 2.33 (s, 3 H, SAc), 2.09 (s, 3 H, Ac), 2.04 (s, 3 H, Ac), 2.03 (s, 3 H, Ac), 2.01 (s, 3 H, Ac), 1.59–1.52 (m, 4 H, –CH_2_–), 1.37–1.32 (m, 4 H, –CH_2_–); 13C{1H} NMR (CDCl3, 101 MHz) δ 196.0, 170.7, 170.3, 169.4, 169.3, 100.8, 72.9, 71.8, 71.3, 69.0, 68.5, 62.0, 30.6, 29.4, 29.2, 29.0, 28.4, 25.3, 20.8, 20.7, 20.6, 20.6.

#### 2-S-Ethyl-β-D-glucopyranose S-linked maleimide (6)^37^

A solution of 4 (50.0 mg, 111 μmol) in methanol (950 μL) was added to 28% NaOMe-MeOH (50 μL) and then stirred at room temperature for 1 h. Diaion™ SK1B was added to the solution for quenching and filtration and evaporation. The residue was dissolved in methanol (2.22 mL), Et3N (22 μL) and maleimide (53.9 mg, 555 μmol) were added, and the mixture was stirred at room temperature for 1 h. The solution was concentrated, and the residue was purified by silica gel chromatography with 50:1 to 5:1 (v/v) dichloromethane-methanol to give 6 (30.3 mg, 81%): 1H NMR (D2O, 400 MHz) δ 4.30 (d, 1 H, J1,2 = 8.0 Hz, H-1), 3.98–3.90 (m, 2 H, –CH_2_SCH–, –OCH_2_–), 3.75–3.72 (m, 2 H, H-6, –OCH_2_–), 3.53 (dd, 1 H, J5,6a = 6.0 Hz, , J6a,6b = 12.1 Hz, H-6), 3.31–3.25 (m, 2 H, H-3, H-5), 3.21–3.07 (m, 3 H, H-2, H-4, –SCHCH_2_–), 2.90–2.86 (m, 1 H, –CH_2_S–), 2.80–2.75 (m, 1 H, –CH_2_S–), 2.63–2.58 (m, 1 H, –SCHCH_2_–); 13C{1H} NMR (CDCl3, 126 MHz) δ 180.9, 179.7, 102.3, 75.8, 75.5, 72.9, 69.4, 69.0, 60.6, 41.5, 41.4, 37.2, 30.3; MALDI-TOF HRMS m/z: [M + Na] + : Calcd for C12H19N1O8S1 + Na + : 360.0724; Found: 360.0676.

#### 6-S-Hexyl-β-D-glucopyranose S-linked maleimide (7)^37^

A solution of 5 (50.0 mg, 101 μmol) in methanol (950 μL) was added to 28% NaOMe-MeOH (50 μL) and then stirred at room temperature for 2.5 h. Diaion™ SK1B was added to the solution for quenching, filtration, and evaporation. The residue was dissolved in methanol (2.02 mL), Et3N (20 μL) and maleimide (49.0 mg, 505 μmol) were added, and the mixture was stirred at room temperature for 1 h. The solution was concentrated, and the residue was purified by reverse-phase C18 silica gel chromatography with 1:0 to 4:1 (v/v) H2O-CH3CN to give 6 (27.5 mg, 72%): 1H NMR (D2O, 500 MHz) δ 4.32 (d, 1 H, J1,2 = 8.0 Hz, H-1), 3.98–3.93–3.90 (m, 1 H, –CH2SCH–), 3.82–3.78 (m, 2 H, H-6a, –OCH_2_–), 3.61–3.52 (m, 2 H, H-6b, –OCH_2_–), 3.38–3.30 (m, 2 H, H-3, H-5), 3.27–3.10 (m, 3 H, H-2, H-4, –SCHCH_2_–), 2.67–2.57 (m, 3 H, –SCHCH_2_–, –CH_2_S–), 1.52–1.47 (m, 4 H, –CH_2_–), 1.32–1.24 (m, 4 H, –CH_2_–); 13C{1H} NMR (CDCl3, 126 MHz) δ 181.2, 179.9, 102.0, 75.8, 75.7, 73.0, 70.4, 69.5, 60.6, 41.4, 37.2, 30.0, 28.4, 28.1, 27.4, 24.4; MALDI-TOF HRMS m/z: [M + Na] + : Calcd for C16H27N1O8S1 + Na + : 416.1350; Found: 415.1309.

### Statistical analysis

The values are reported as the means ± standard error (n = 6) with six individual data points. Statistical significance (single-sided Student’s t test) is indicated in the figure legends as follows: *p < 0.05. n = 6 for statistical analysis^22,23,25,26,34–36^.

### Ethical approval

The study is reported in accordance with ARRIVE guidelines. All mouse experiments were performed in accordance with the ethical guidelines for animal care of National Center for Geriatrics and Gerontology, and the experimental protocols were approved by the Animal Care Committee of National Center for Geriatrics and Gerontology^22,23,25,26,34–36^.

## Supplementary Information

Supplementary Information.

## Data Availability

Requests for data and materials should be addressed to TI (timai@ncgg.go.jp).
