# Supplementary material for: UDP-glucose, cereblon-dependent proinsulin degrader
Source: Sci Rep. 2022 Aug 26;12:14568. doi: 10.1038/s41598-022-18902-5 (PMC9418190; doi:10.1038/s41598-022-18902-5)
Supplement: Supplementary file 1 — Supplementary Information. [file 41598_2022_18902_MOESM1_ESM.pdf]

Supplemental information for

UDP-glucose, cereblon-dependent proinsulin degrader.

This pdf file includes:

Abbreviations

Supplementary Figure S1 to 6

Abbreviations

CRBN, cereblon

CRL4, cullin ring ubiquitin ligase 4

CUL4, cullin 4

DDB1, damage-specific DNA binding protein 1

ER, endoplasmic reticulum

ERAD, ER-associated protein degradation

ERRS, ER-retention signal

GCK, glucokinase

GLUT, glucose transporter

HOMAβ, homeostatic model assessment of β cells

HRD1, HMG-CoA reductase degradation 1

IC_{50}, half maximal (50%) inhibitory concentration

IP, immunoprecipitation

MODY, maturity-onset diabetes of the young

TCA cycle, tricarboxylic acid cycle

UGGT, UDP-glucose:glycoprotein glucosyltransferase

UPR, unfolded protein response

WB, Western blot

WT, wild type

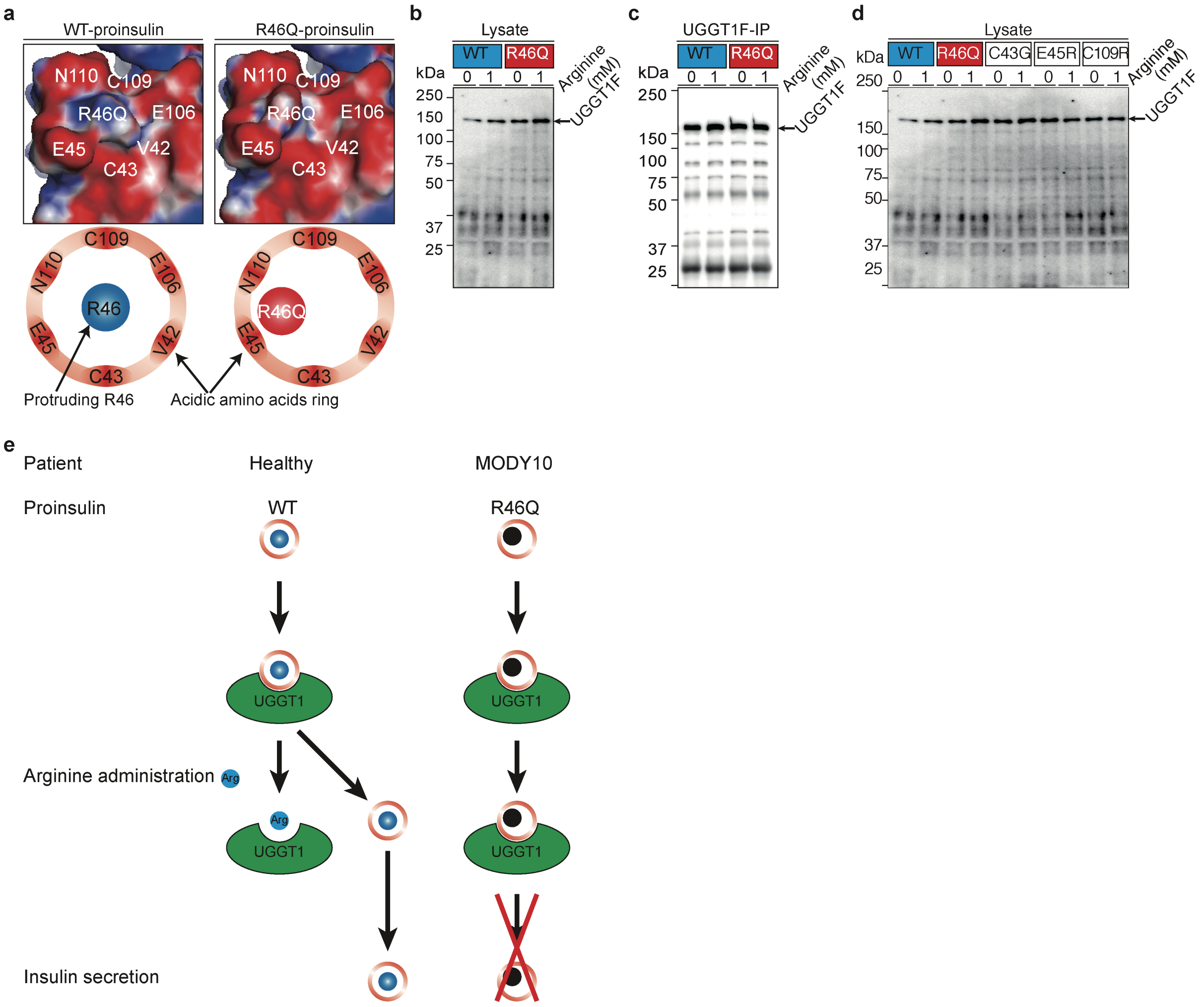

Supplementary Figure S1. R46 residue of proinsulin is involved in arginine-induced insulin secretion (Supplementary data for Fig. 1).

(**S1a**) The predicted structure of R46 (WT) and R46Q proinsulin.

(**S1b** and **c**) Intracellular proinsulin and UGGT1 were analyzed by WB (**c** and Fig. **S1b**). Proinsulin bound to UGGT1 was assessed by IP of UGGT1 followed by IP-WB (Fig. 1**d** and Fig. **S1c**).

(**S1d**) Proinsulin was immunoprecipitated and blotted for UGGT1 (**c**, **d**, Fig. **S1b**, **S1c** and **S1d**).

(**S1e**) Possible model of arginine-induced R46Q secretion.

WT-insulin binds to UGGT1, and is retained in the ER. Arginine administration releases proinsulin from UGGT1 in a competitive manner.

On the other hands, R46Q proinsulin binds to UGGT1 like WT-proinsulin in the absence of

arginine. Arginine administration has no effect on the R46Q and UGGT1 interaction, leading to less induced R46Q secretion by arginine.

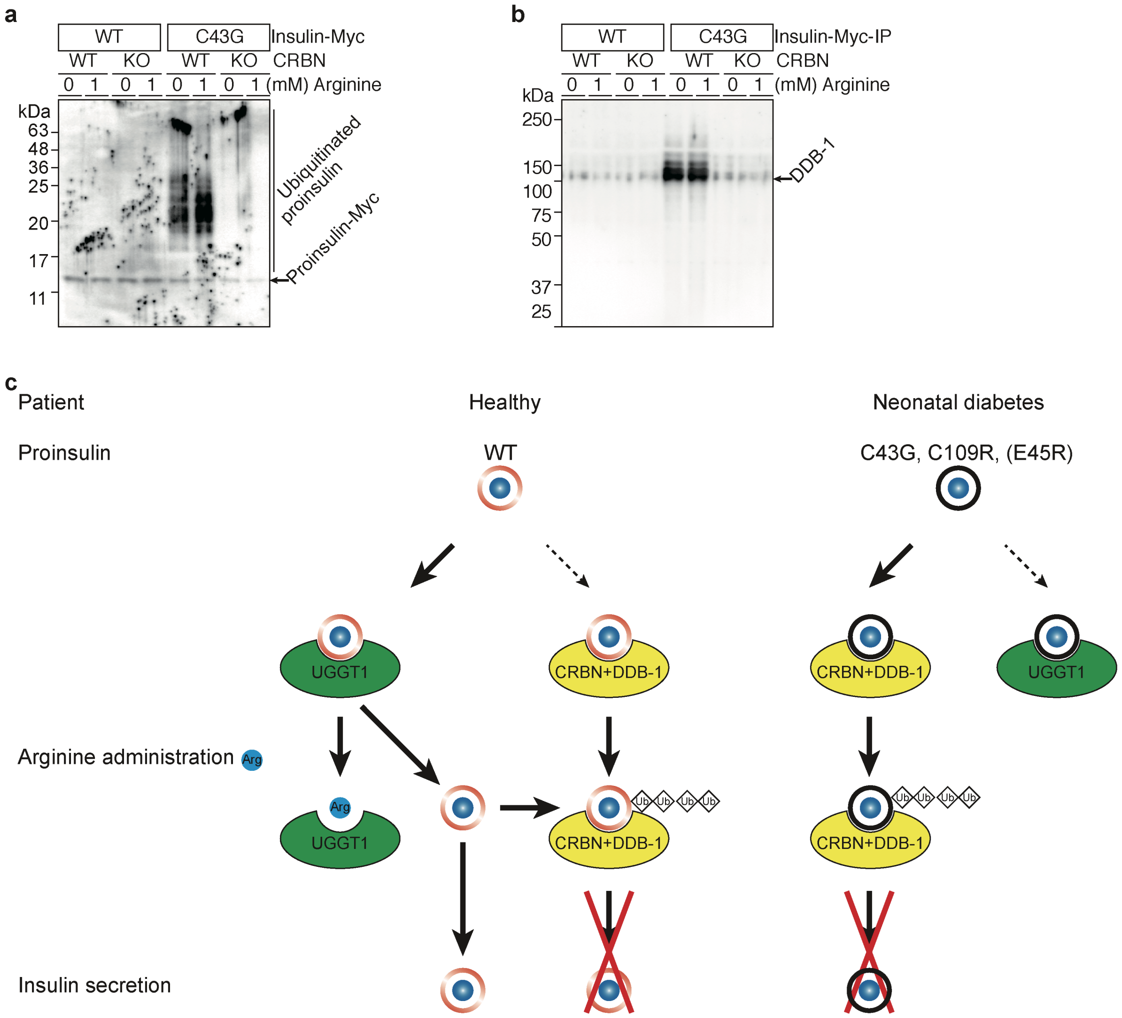

Supplementary Figure S2. Impaired UGGT1-interaction in C43G-neonatal diabetes variant (Supplementary data for Figure 2).

(**S2a** and **S2b**) Ubiquitination of proinsulin^{C43G} protein and association with DDB1 was observed only in CRBN^{WT} cells (Fig. 2**d**, **e**, Fig. **S2a** and **S2b**).

(**f**) Summary of interaction of insulin (R46Q and C43G variants) with UGGT1 and cereblon in the presence and absence of arginine.

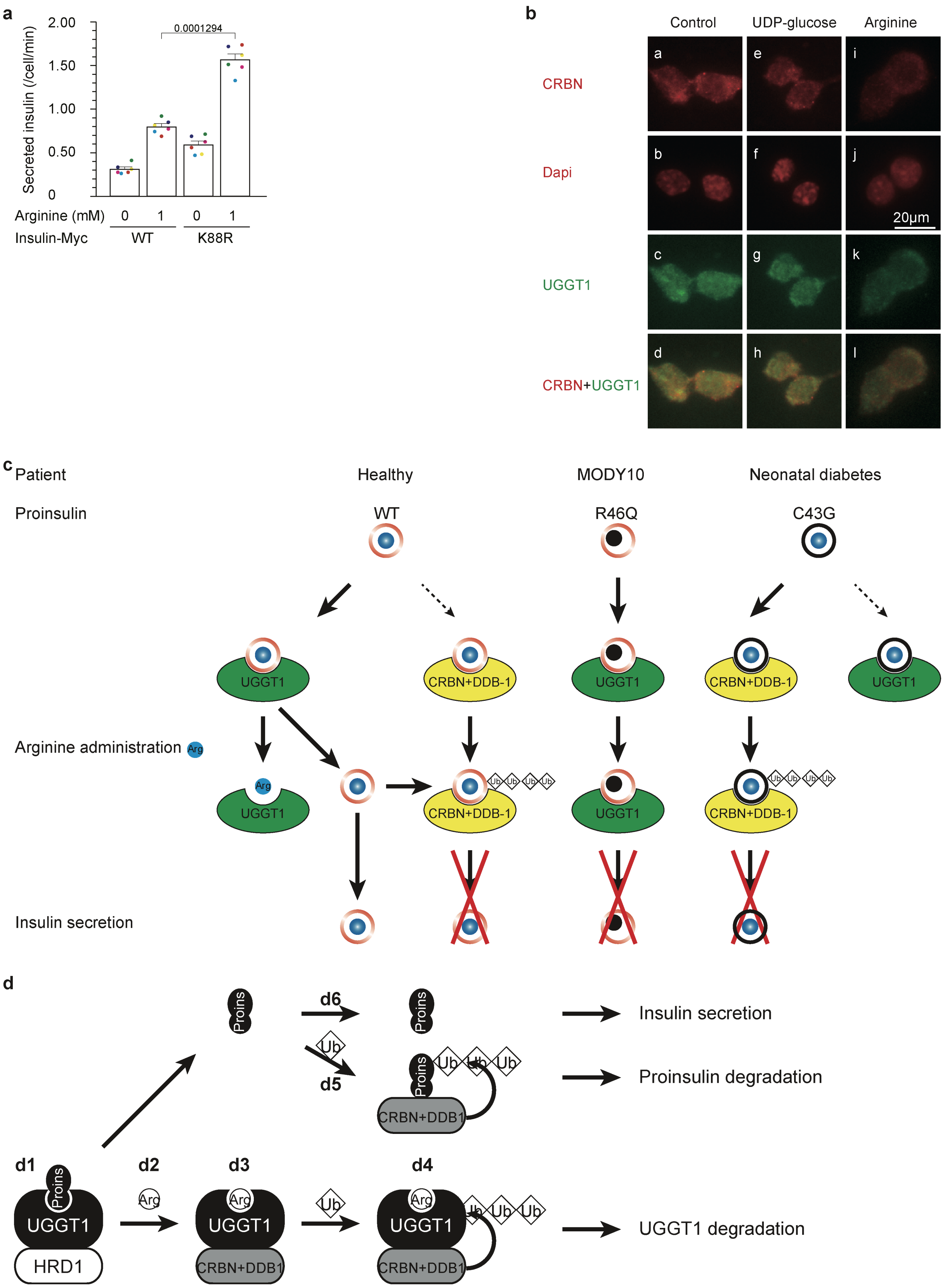

Supplementary Figure S3. Arginine switches proinsulin associated E3 ubiquitin ligases from HRD1 to cereblon. (Supplementary data for Fig. 3).

(**S3a**) Higher amount of secreted insulin^{K88R} with/without arginine.

(**S3b**) Cereblon co-locates with UGGT1 in the ER. The NIT1 cells treated with control, UDP-glucose, and arginine were visualized with anti-CRBN (red color), Dapi (red color), UGGT1 (green color). Co-localized cereblon and UGGT1 merged into orange color.

(**S3b**) Possible model of arginine-induced WT, R46Q and C43G-insulin secretion.

(Healthy) WT-proinsulin; in the absence or low concentration of arginine, WT-proinsulin binds to UGGT1 mainly. Arginine administration releases proinsulin from UGGT1. Free proinsulin partially binds to cereblon and DDB1 for ubiquitination, and the rest free proinsulin will be secreted.

(MODY10) R46Q-proinsulin; in the absence of arginine R46Q binds to UGGT1, although arginine administration has no effect on the R46Q-UGGT1 interaction, leading less arginine-induced R46Q secretion.

(Neonatal diabetes) C43G-proinsulin; in the absence and presence of arginine C43G binds mainly to CRBN and DDB1 and is ubiquitinated.

(**S3c**) Possible model of arginine-induced insulin secretion with inhibition of proinsulin ubiquitination and degradation.

(**S3d**) Possible model of arginine-induced insulin secretion

(d1) In the absence or low concentration of arginine, proinsulin binds to UGGT1 and HRD1.

(d2) Arginine administration triggers proinsulin releasing from UGGT1 and HRD1 complex.

(d3) Proinsulin-free UGGT1 is also release from HRD1 and binds to arginine, CRBN and DDB1.

(d4) This arginine-UGGT1-CRBN-DDB1 complex leads to UGGT1 ubiquitination.

(d5) A half of free proinsulin binds to CRBN and DDB1 with unknown endogenous ligand X, and is ubiquitinated and degraded.

(d6) The rest half proinsulin is secreted.

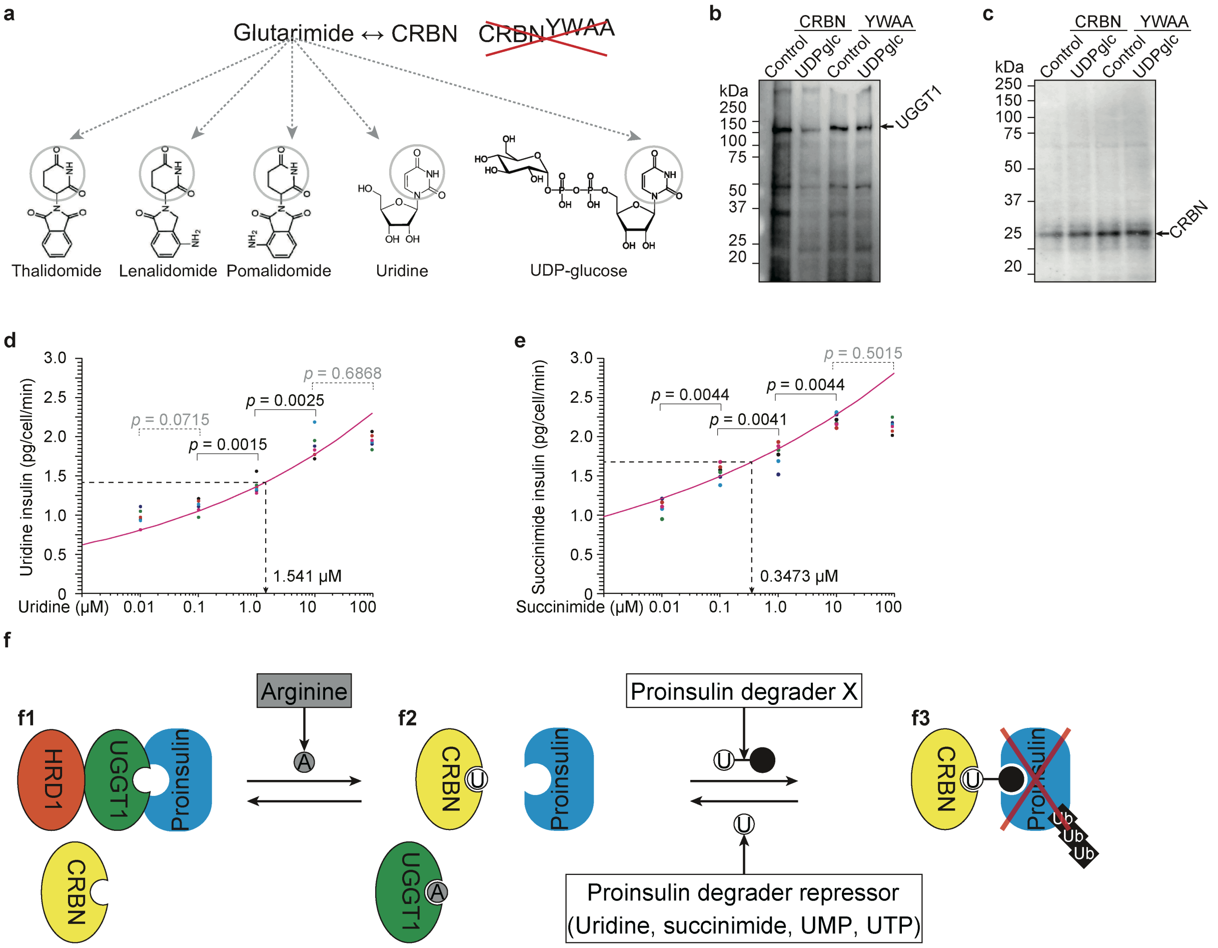

Supplementary Figure S4. Uridine and succinimide protect proinsulin degradation from cereblon and stimulate insulin secretion from 3h to 24h. (Supplementary data for Fig. 4).

(**S4a**) Glutarimide base in IMiDs, uridine and UDP-glucose.

(**S4b** and **c**) UDP-glucose and cereblon dependent UGGT1 ubiquitination and degradation, although $CRBN^{YWAA}$ mutant was resistant to UDP-glucose dependent UGGT1 ubiquitination and degradation.

(**S4d**) Uridine stimulates insulin secretion. IC_{50} = 1.541 μM

(**S4e**) Succinimide stimulates insulin secretion. IC_{50} = 0.3473 μM

(**S4f**) Possible model insulin secretion.

(f1) In the absence or low concentration of arginine, proinsulin binds to UGGT1 and HRD1.

(f2) Arginine binds to UGGT1, and proinsulin releases from UGGT1. Uridine binds to cereblon to mask the interaction with proinsulin

(f3) Proinsulin degrader X interacts with cereblon and proinsulin, and proinsulin was

ubiquitinated and degraded.

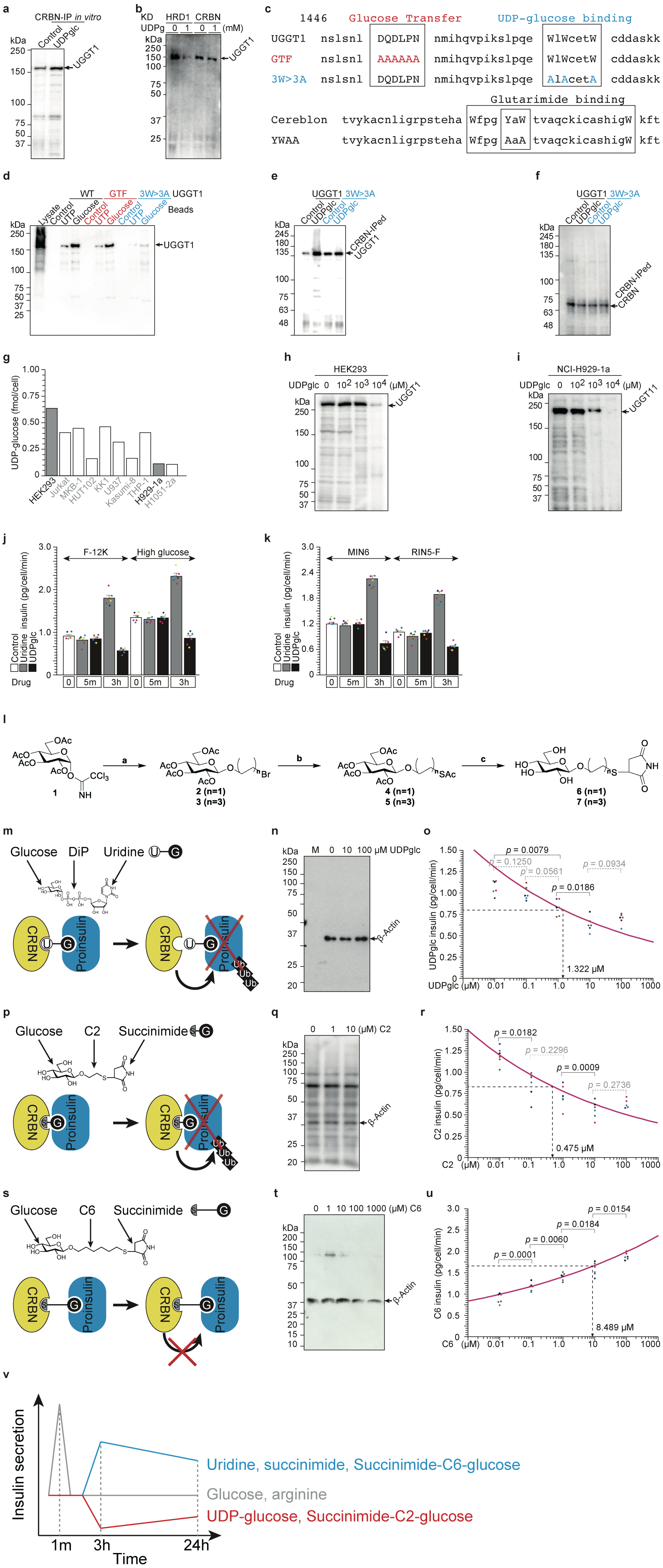
a
CRBN-IP in vitro
Control
UDPglc
UGGT1
b
HRD1
CRBN
UDPg
(mM)
UGGT1
c
1446
Glucose Transfer
UDP-glucose binding
UGGT1 nslsnl DQDLPN nmihqvpikslpqe WlWcetW cddaskk
GTF nslsnl AAAAAA nmihqvpikslpqe WlWcetW cddaskk
3W>3A nslsnl DQDLPN nmihqvpikslpqe AlAcetA cddaskk
Glutarimide binding
Cereblon tvykacnligrpsteha Wfpg YaW tvaqckicashigW kft
YWAA tvykacnligrpsteha Wfpg AaA tvaqckicashigW kft
d
WT
GTF
3W>3A
UGGT1
Lysate
Control
UTP
Glucose
Beads
e
UGGT1 3W>3A
CRBN-IPed
f
CRBN
g
UDP-glucose (fmol/cell)
HEK293
Jurkat
MKB-1
HUT102
KK1
U937
Kasumi-8
THP-1
H929-1a
H1051-2a
h
HEK293
UDPglc
i
NCI-H929-1a
j
F-12K
High glucose
insulin (pg/cell/min)
Control
Uridine
UDPglc
Drug
k
MIN6
RIN5-F
l
m
Glucose
DiP
Uridine
CRBN
Proinsulin
n
M 0 10 100 μM UDPglc
β-Actin
o
UDPglc insulin (pg/cell/min)
p = 0.0079
p = 0.1250
p = 0.0561
p = 0.0934
p = 0.0186
1.322 μM
p
Glucose
C2
Succinimide
q
0 1 10 (μM) C2
r
C2 insulin (pg/cell/min)
p = 0.0182
p = 0.2296
p = 0.0009
p = 0.2736
0.475 μM
s
Glucose
C6
Succinimide
t
0 1 10 100 1000 (μM) C6
u
C6 insulin (pg/cell/min)
p = 0.0154
p = 0.0184
p = 0.0060
p = 0.0001
8.489 μM
v
Insulin secretion
Uridine, succinimide, Succinimide-C6-glucose
Glucose, arginine
UDP-glucose, Succinimide-C2-glucose
1m
3h
24h
Time

Supplementary Figure S5. UDP-glucose is endogenous proinsulin protein degrader through cereblon (Supplementary data for Fig. 5).

(**S5a**) Cereblon bound to UGGT1 directly *in vitro*, and UDP-glucose induced the interaction between cereblon and UGGT1.

(**S5b**) UDP-glucose dependent UGGT1 ubiquitination and degradation was cereblon-dependent manner.

(**S5c**) Protein sequences of two UGGT1 mutant protein of GTF and 3W>3A.

(**S5d**) UGGT1-WT and GTF mutant bound to UTP and glucose, although UGGT1-3W>3A mutant protein did not bind to neither UTP nor glucose.

(**S5e** and **f**) Both $UGGT1^{WT}$ and $UGGT1^{3W>3A}$ protein bound to cereblon without UDP-glucose. UDP-glucose administration induced cereblon-interaction of $UGGT1^{WT}$, did not induce that of $UGGT1^{3W>3A}$.

(**S5g-i**) Endogenous UDP-glucose induced UGGT1 degradation. Intracellular endogenous UDP-glucose concentration in various cell lines (**g**). Ten mM UDP-glucose administration induced UGGT1 degradation in HEK293 cells (**h**). One mM UDP-glucose administration induced UGGT1 degradation in NCI-H929-1a cells (**i**).

(**S5j**) Uridine-induced insulin secretion, but UDP-glucose-reduced insulin secretion in F-12K medium with normal (7 mM) or high (24.9 mM) glucose concentration.

(**S5k**) Uridine-induced insulin secretion, but UDP-glucose-reduced insulin secretion in MIN6 and RIN5-F cells.

(**S5l**) Synthesis of glucose conjugated maleimide by thiol–ene click reaction

Synthesis of glucose conjugated maleimide. Reagents and conditions: (a) 2-bromoethanol, BF_3-Et_2O, CH_2Cl_2, −40 °C, 30 min, 66% (for 2); 6-bromohexanol, BF_3-Et_2O, CH_2Cl_2, −40 °C, −30 min, −20 °C, 30 min, and then 0 °C, 30 min, 66% (for 3); (b) AcSH, K_2CO_3, DMF, r.t., 12 h, 99% (for 4); AcSH, K_2CO_3, DMF, r.t., 17.5 h, 83% (for 5); (c) (i) NaOMe−MeOH, r.t., 1 h, (ii) Maleimide, Et_3N, MeOH, r.t., 1 h, 81 % (for 6); (i) NaOMe−MeOH, r.t., 2.5 h, (ii) Maleimide, Et_3N, MeOH, r.t., 1 h, 72 % (for 7).

(**S5m-o**) UDP-glucose degrades proinsulin at IC_{50} = 1.322 μM

(**S5p-r**) Suciinimide-C2-glucose degrades proinsulin at IC_{50} = 0.475 μM

(**S5s-u**) Suciinimide-C6-glucose protects proinsulin degradation at IC_{50} = 8.489 μM

(**S5v**) Sustainable insulin secretion

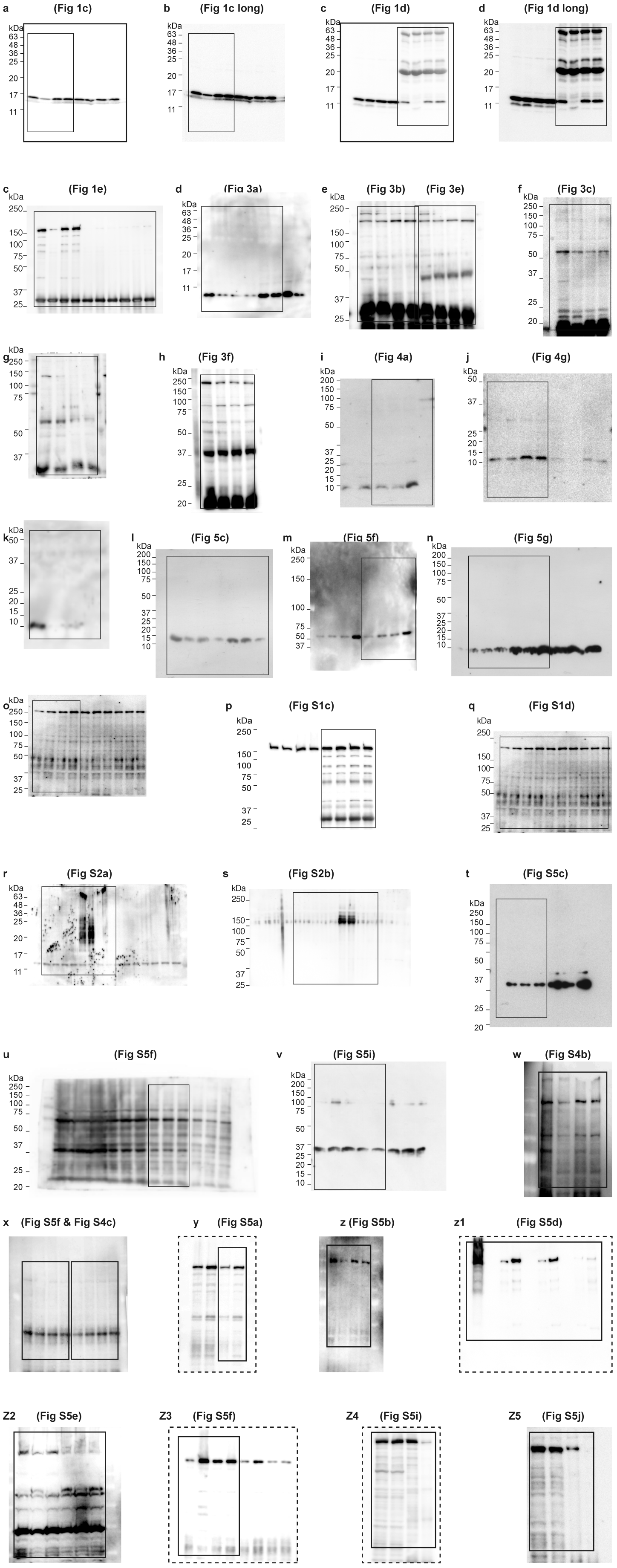

a (Fig 1c)
b (Fig 1c long)
c (Fig 1d)
d (Fig 1d long)
c (Fig 1e)
d (Fig 3a)
e (Fig 3b) (Fig 3e)
f (Fig 3c)
h (Fig 3f)
i (Fig 4a)
j (Fig 4g)
l (Fig 5c)
m (Fig 5f)
n (Fig 5g)
p (Fig S1c)
q (Fig S1d)
r (Fig S2a)
s (Fig S2b)
t (Fig S5c)
u (Fig S5f)
v (Fig S5i)
w (Fig S4b)
x (Fig S5f & Fig S4c)
y (Fig S5a)
z (Fig S5b)
z1 (Fig S5d)
Z2 (Fig S5e)
Z3 (Fig S5f)
Z4 (Fig S5i)
Z5 (Fig S5j)
kDa

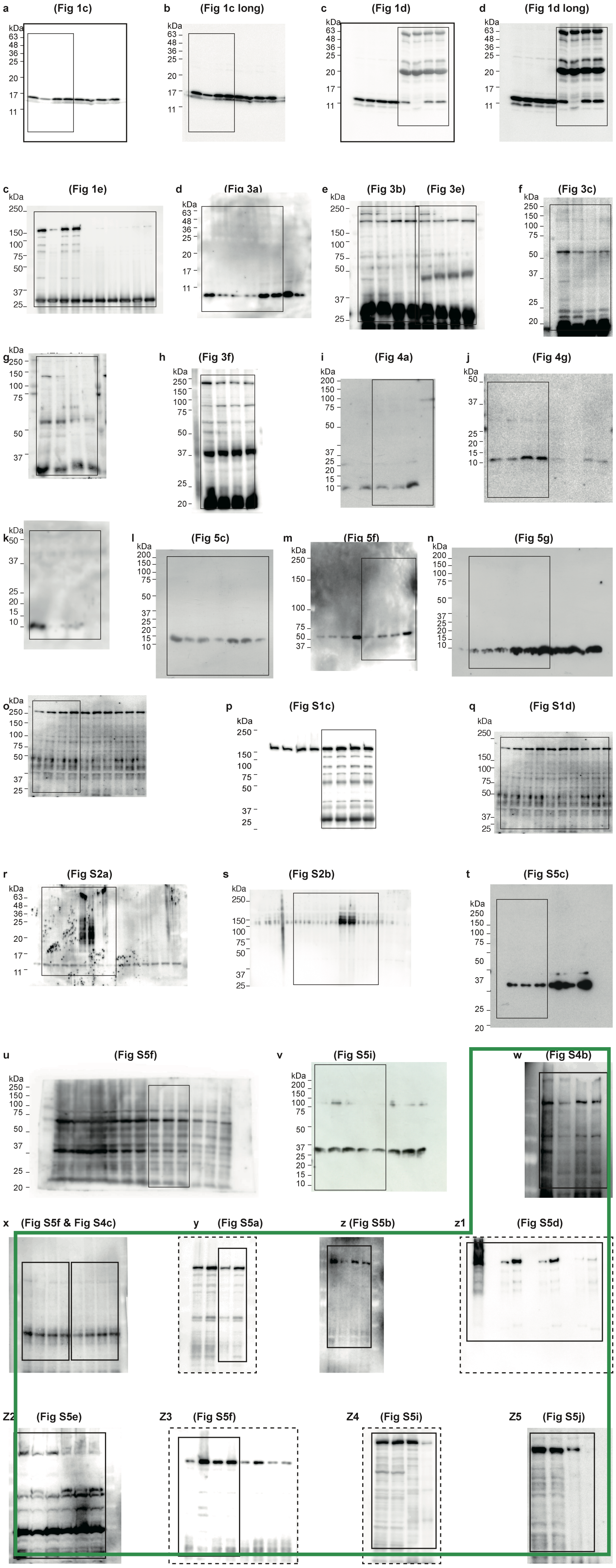
a (Fig 1c)
b (Fig 1c long)
c (Fig 1d)
d (Fig 1d long)
c (Fig 1e)
d (Fig 3a)
e (Fig 3b) (Fig 3e)
f (Fig 3c)
g
h (Fig 3f)
i (Fig 4a)
j (Fig 4g)
k
l (Fig 5c)
m (Fig 5f)
n (Fig 5g)
o
p (Fig S1c)
q (Fig S1d)
r (Fig S2a)
s (Fig S2b)
t (Fig S5c)
u (Fig S5f)
v (Fig S5i)
w (Fig S4b)
x (Fig S5f & Fig S4c)
y (Fig S5a)
z (Fig S5b)
z1 (Fig S5d)
Z2 (Fig S5e)
Z3 (Fig S5f)
Z4 (Fig S5i)
Z5 (Fig S5j)
kDa

Figure S6 Source data
